# Comparison of Three Different Doses of Dexmedetomidine for Attenuation of the Pressor Response to Laryngoscopy and Intubation by Assessment of Haemodynamic Parameters and Plasma Catecholamine Levels Under Bi-spectral Index Guided Anaesthesia - A Randomised Double Blind Controlled Study

**DOI:** 10.4274/TJAR.2026.252245

**Published:** 2026-04-15

**Authors:** Michell Gulabani, Medha Mohta, Nitesh Kumar, Diwesh Chawla, Seema Garg, Shashank Tripathi, Abhay Singh Chauhan

**Affiliations:** 1 University Faculty of Medical Sciences and GTB Hospital, Department of Anaesthesiology and Intensive Care, Delhi, India; 2 University College of Medical Sciences and GTB Hospital, Departent of Multi-disciplinary Research Unit, Delhi, India; 3 University College of Medical Sciences and GTB Hospital, Department of Biochemistry, Delhi, India; 4 University College of Medical Sciences and GTB Hospital, Department of Biostatistics and Medical Informatics, Delhi, India

**Keywords:** Bradycardia, catecholamines, dexmedetomidine, endotracheal intubation, hypotension, propofol

## Abstract

**Objective:**

Dexmedetomidine has been studied for attenuation of laryngoscopy and intubation response with varied results, however the depth of anaesthesia and plasma catecholamine levels have not been measured. Considering the lacunae, this study was planned with three doses of dexmedetomidine: 0.5, 0.75 and 1 μg kg^-1^ under bi-spectral index monitoring and plasma catecholamine assessment.

**Methods:**

One hundred sixty-eight consenting adult patients of either sex were divided into 3 groups (56 each) to receive I/V dexmedetomidine 0.5, 0.75 or 1 μg kg^-1^ prior to induction. A baseline sample of catecholamines was taken and study drug was infused over 10 minutes. Thereafter, standard anaesthesia induction followed and haemodynamic parameters were noted at designated time intervals. Another sample of catecholamines was drawn at 3 minutes after intubation. The primary outcome was to compare the change in heart rate and systolic blood pressure. Secondary outcomes included: change in catecholamine levels, sedation scores, propofol dose and adverse events.

**Results:**

All doses of dexmedetomidine successfully obtunded the haemodynamic response; however, no significant difference was seen on inter-group comparison (*P* >0.05). A significant fall in nor-adrenaline values compared with baseline was noted in all groups, without any significant difference among groups for both catecholamines. Sedation scores reduced from baseline in all groups without any difference on inter- group comparison. Statistically significant reduction in propofol requirement and higher incidence of bradycardia with 1 µg kg^-1^ (*P*=0.014) were observed.

**Conclusion:**

0.5 μg kg^-1^ of dexmedetomidine can be used for pressor response attenuation as the incidence of bradycardia was higher with 1 μg kg^-1^ and 0.75 μg kg^-1^ had no added advantage.

**Main Points**

• Dexmedetomidine: 0.5, 0.75,1 μg kg^-1^ were compared for pressor response attenuation.

• Haemodynamic response were successfully obtunded by all doses of dexmedetomidine.

• The three doses did not differ in haemodynamic parameters, adrenaline, nor-adrenaline levels.

• Reduction in propofol requirement but higher incidence of bradycardia with the 1 µg kg^-1^ dose.

• Dexmedetomidine: 0.5 µg kg^-1^ is beneficial, optimal, cost-effective less side effects.

## Introduction

Laryngoscopy and intubation are noxious stimuli that evoke numerous undesirable effects, such as tachycardia, hypertension, cardiac dysrhythmias, and increased plasma catecholamine levels by stimulating oropharyngeal and laryngeal structures.^1^ The onset of this intubation response occurs within 5 seconds of airway manipulation, peaks at 1-3 minutes, and stabilizes by 5-10 minutes.^2^ Most healthy patients tolerate this transitory phenomenon, but it can be life threatening for cardiac, neurosurgical and elderly patients who might experience myocardial ischemia during this vulnerable time.^3, 4^

A variety of pharmacological methods have been utilized for attenuation of the response at intubation which include lignocaine,^5^ beta blockers,^6^ calcium channel blockers,^7^ nitroglycerine,^8^ opioids,^9^ and alpha adrenergic 2 agonists like dexmedetomidine.^10, 11, 12^

Dexmedetomidine is superior to other agents because of its multiple actions, which include conscious sedation without respiratory depression, anaesthetic-sparing, analgesic, anxiolytic, anti-sialogogue, antiemetic, and antitussive effects.^13^

Obtundation of the pressor response is one of the most frequently topics in the field of anaesthesiology. Anaesthesiologists have continually sought an agent that effectively suppresses adverse cardiovascular insults while providing a maximal safety margin.

In many of the studies on dexmedetomidine for pressor- response obtundation available in the literature, the depth of anaesthesia has not been monitored, which is vital in this regard, as inadequate depth is one of the precipitating factors for intubation response.^2, 10, 11^ The plasma adrenaline and nor-adrenaline levels, the biochemical markers of stress response, have not been studied across different doses of dexmedetomidine in this context, and researchers have cited these as limiting factors.^12, 14, 15^

Hence, considering the existing lacunae in the literature, the present study was designed to determine the optimal dose of dexmedetomidine to attenuate the pressor response by comparing three doses: 0.5 μg kg^-1^, 0.75 μg kg^-1^, and 1 μg kg^-1^ in patients scheduled for elective operative procedures under general anaesthesia (GA) with bi-spectral index (BIS) monitoring. The primary objective was to study and compare the changes in heart rate (HR) and systolic blood pressure (SBP) following endotracheal intubation. The secondary objectives were to compare changes in plasma adrenaline and nor-adrenaline levels, Modified Observer’s Assessment of Alertness/Sedation Scale (MOAS) scores, the total requirement of propofol for anaesthesia induction, and the incidence of perioperative complications among the three study groups.

## Methods

This prospective randomised, double‑blind, controlled study was conducted in a tertiary care teaching hospital after written informed consent was obtained from the participants. It was approved by the Delhi University Faculty of Medicine, Human Research (IEC-HR) Institutional Ethics Committee, (approval no: IECHR-2023-61-12-R2, date: 02.11.2023). Registered with the Clinical Trials Registry‑India (CTRI) under registration CTRI/2023/12/060514 (www.ctri.nic.in).The patients were randomly assigned to one of the three study groups (56 participants each) using a computer-generated random number table. Allocation concealment was performed using sequentially numbered opaque sealed envelopes.

A complete pre-anaesthetic checkup and appropriate investigations were conducted in accordance with hospital protocol. A minimum fasting period of 8 hours prior to surgery was ensured for all patients.

One hundred sixty-eight consenting adult patients aged 18-60 years of either sex, American Society of Anaesthesiologists Physical Status (ASA-PS) Grade I and II, undergoing elective surgical procedures under GA with endotracheal intubation were enrolled. Patients with a history suggestive of diabetes mellitus, asthma, uncontrolled hypertension morbid obesity, altered liver or kidney function, or progressive neurological disease, as well as those who were pregnant, were excluded from the study. Those with anticipated difficult airway, laryngoscopy and intubation time of more than 15 seconds, or more than one attempt at laryngoscopy and endotracheal intubation were also excluded.

### Anaesthesia Technique

An 18 G intravenous (IV) cannula was inserted in the preoperative holding area, and a venous blood sample for plasma adrenaline and nor-adrenaline was drawn for the first time. Subsequently, a Ringer’s lactate drip was administered to all study participants at a rate adjusted to 5-6 mL kg^-1^ per hour.

In the operating room, all standard monitors, including non-invasive blood pressure on the upper arm, oxygen saturation (SpO_2_) probe, electrocardiogram, and BIS electrodes on the forehead, were applied to the patient. The baseline parameters [Ta] including HR, SBP, diastolic blood pressure (DBP), mean blood pressure (MBP), SpO_2_ and BIS were recorded. The MOAS sedation scale, which classifies alertness and sedation using a score ranging from 0 to 6 (0 denotes deep sedation and 6 denotes an agitated state), was employed for all patients in each group.^16^

The study drug was prepared by an individual who was not actively involved in the research, and both the patient and the investigator were blinded to the group allocation. The patients in Group D1, Group D2, and Group D3 were given IV dexmedetomidine as loading doses of 0.5 μg kg^-1^, 0.75 μg kg^-1^, and 1 μg kg^-1^, respectively, diluted in normal saline up to 50 ml and administered over 10 minutes prior to induction. During IV infusion of the study drug, if the SpO_2 _dropped below 95%, oxygen was supplemented by a simple face mask at the rate of 5-6 liters/minute, and this was noted as an episode of desaturation. On completion of the study drug infusion, all the parameters [Tb], along with the MOAS score and BIS, were noted. Anaesthesia was induced with inj. fentanyl 2 μg kg^-1^ and titrated doses of inj. propofol administered from a syringe loaded with 2 mg kg^-1^. The failure to respond to verbal commands was considered as the end point of induction, at which all the study parameters and BIS were recorded again [Tc].

After, administration of 0.1 mg kg^-1^ of inj. vecuronium bromide and ventilating the patient for 3 minutes, just before performing laryngoscopy, all parameters [Td] were noted. A senior anaesthesiologist performed laryngoscopy and tracheal intubation with an endotracheal tube of appropriate size. The study parameters were noted at 1 minute [Te], 2 minutes [Tf], 3 minutes [Tg], 5 minutes [Th], and 7 minutes [Ti] following intubation in all three groups. Anaesthesia was maintained with sevoflurane in oxygen and nitrous oxide (50:50) at a flow of 2 liters per minute. Throughout, the BIS values were maintained between 40-60 by adjusting the dial settings of the sevoflurane vaporizer. At time Tg, the venous blood sample for plasma adrenaline and nor-adrenaline was drawn a second time. After the last recording of the study parameters, i.e., Ti, the study was completed. The total propofol requirement for induction was recorded in all study groups.

The time points: on arrival in the operating room, after study drug infusion, following anaesthesia induction, just before laryngoscopy and intubation and then at 1, 2, 3, 5 and 7 minutes after endotracheal intubation were the designated intervals where HR, SBP, DBP, MBP, SpO_2_ and BIS were noted. The sample for catecholamine analysis was drawn twice: once in the preoperative area as a baseline value, and once on the operating room table 3 minutes after endotracheal intubation.

IV ondansetron was administered to patients 30 minutes prior to the end of the surgery. Upon completion of the surgical procedure, neuromuscular blockade was reversed with neostigmine 0.05 mg kg^-1^ and glycopyrrolate 0.01 mg kg^-1^. The tracheal tube was removed after adequate spontaneous ventilation was established and protective airway reflexes had returned.

Hypotension was defined as a >30% fall in SBP from the baseline value, bradycardia as HR<60 beats/minute, and desaturation as SpO_2 _<95%>. Hypotension was treated by reducing the inhalational agent concentration by 50% when the BIS value was on the lower side of the required range (40-60), and/or by administering IV mephentermine 6 mg; bradycardia was treated with IV atropine 0.6 mg. The maintenance of anaesthetic depth was always ensured by keeping BIS values between 40-60.

### Outcomes

The primary outcome was to study and compare changes in HR and SBP from baseline parameters (i.e., before administration of the study drug) to those after dexmedetomidine administration in all three groups at designated time intervals, namely, 1, 2, 3, 5, and 7 minutes after intubation, while maintaining BIS values between 40-60. The secondary outcome parameters were: the change in plasma adrenaline and nor-adrenaline levels from baseline to values following administration of three different doses of dexmedetomidine, measured 3 minutes after endotracheal intubation; the change in the MOAS score, recorded at baseline and after completion of study drug infusion (10 minutes); the total requirement for propofol for anaesthesia induction; and the incidence of perioperative complications including hypotension, bradycardia, or desaturation.

### Sample Size Calculation

The sample size was calculated based on the basis of the study by Sebastian et al where the authors observed that HR and SBP at 3 minutes post intubation in dexmedetomidine 0.5 μg kg^-1^ group were 83.73±4.95 beats/minute and 129.87±9.75 mmHg respectively and in dexmedetomidine 0.75 µg kg^-1^ were 80.83±5.40 beats/minute and 124.67±8.41 mmHg respectively.^17^ Using these values as references, the minimum required sample size, with 80% study power and a 5% level of significance, was 168 patients, with 56 patients in each study group.

### Statistical Analysis

The statistical analysis was carried out using the SPSS version 25.0. categorical data were presented as number and percentage (%) and continuous data as mean ± standard deviation and median [interquartile range (IQR)]. The Kolmogorov-Smirnov test was used to assess the normality of the data. Non-parametric tests were employed if the assumption of normality was rejected. Paired data at two time points were compared using the paired t-test or Wilcoxon signed-rank test. Quantitative variables were compared using one-way ANOVA or Kruskal-Wallis test among the three groups, followed by a post-hoc test if a significant result was obtained. Repeated-measures ANOVA or Friedman’s ANOVA was used to compare parameters assessed at multiple time points. Qualitative variables were analysed using the chi-square test or Fisher’s exact test. A *P* value of <0.05 was considered statistically significant.

## Results

**Figure 1:**
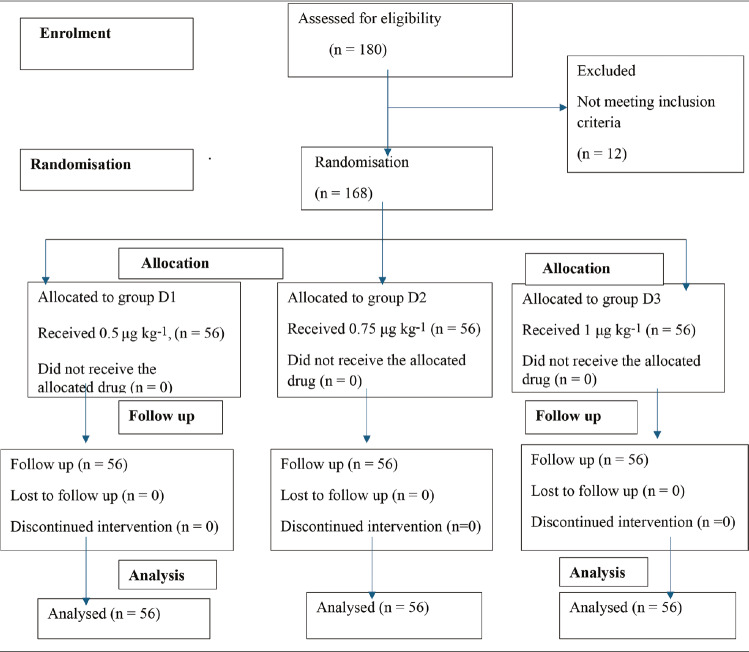
Consolidated standards of reporting trials (CONSORT) flow diagram.

The CONSORT flow diagram is shown in Figure 1. The demographic variables and ASA-PS distributions were comparable across the three study groups (Table 1). The most frequent surgical procedure performed was laparoscopic cholecystectomy, followed by mastectomy and thyroidectomy.

Haemodynamic HR was significantly lower compared with baseline values within each group (D1, D2, and D3) at all time intervals except at 1 minute after intubation (Table 2). Similarly, a statistically significant decrease in SBP compared with baseline levels was observed in the D1 and D3 groups at all time points except at one minute following intubation. However, a decrease in SBP compared with baseline values was observed at every designated time point in the D2 group (Table 3). On inter group comparison, HRs at the end of the study drug infusion in group D3 were significantly lower than those in groups D1 and D2 (Table 2). There were no intergroup differences in HR at any other time intervals. Intergroup comparison showed that SBP readings did not differ between the three groups at any time point (Table 3).

**Catecholamines: **No significant differences were observed in the values of adrenaline and nor-adrenaline among the three dexmedetomidine dose groups at baseline and 3 minutes after intubation. However, no increase was seen in adrenaline or nor-adrenaline levels; rather, nor-adrenaline values were significantly lower at 3 minutes after intubation compared with baseline values within each group, while adrenaline levels remained near baseline values (Tables 4a and 4b). In of the wide IQR, the percent change from baseline in the values of adrenaline and nor-adrenaline was calculated (Table 5). No significant differences were found between groups for these values.

**Supplementary outcomes:** The MOAS scores were comparable between the groups (*P *>0.05), although a statistically significant reduction was seen in them after study drug infusion compared with baseline values within all groups. A significantly higher incidence of bradycardia was observed in the 1 μg kg^-1^ group (7 patients out of 56) compared with the other two groups (0.5 µg kg^-1^ and 0.75 µg kg^-1^), in each of which 1 patient out of 56 was affected (*P*=0.014). A comparable incidence of hypotension (9, 12, and 8 patients in D1, D2, and D3 groups, respectively; *P*=0.581) was observed, and no desaturation event occurred in any group. A significantly lower propofol dose requirement with the 1 μg kg^-1^ dose of dexmedetomidine, compared with both other groups, was observed (*P *<0.05) (D1 vs D3 *P* <0.05, D2 vs D3* P*=0.031). A statistically significant, steady decline in BIS values from baseline to values after completion of study drug infusion and at induction was observed in all three groups (*P *<0.05). However, no significant difference was found in BIS values between the groups.

## Discussion

In the present study, all three dexmedetomidine doses, i.e., 0.5 µg kg^-1^, 0.75µg kg^-1^, and 1 µg kg^-1^, successfully blunted the haemodynamic response and the catecholamine rise to endotracheal intubation. Sedation scores and BIS values were comparable between the groups, but were reduced from baseline values within each group. The dose of 1 µg kg^-1^ reduced the propofol requirement, but resulted in a higher incidence of bradycardia than the lower doses.

Dexmedetomidine has been shown to be effective in attenuating the pressor response to laryngoscopy and intubation.^18, 19, 20^ It has been compared in several doses ranging from 0.5 μg kg^-1^ to 1 μg kg^-1^ with varied results.^4,5,10, 11, 12,14,15,17^

The results of the present study are in concordance with those of some earlier studies, where a comparable obtundation in intubation response was seen with two different doses of dexmedetomidine, i.e., 0.5 and 1 µg kg^-1^ and 0.5 and 0.75 μg kg^-1^.^2, 11, 17^ Contrary to our observations, a few researchers have found that a higher dose of dexmedetomidine (1 μg kg^-1^) is more effective in attenuating the pressor response at intubation.^5, 10, 15^

However, all the above-mentioned studies were affected by one or more limitations, including a small sample size, lack of plasma catecholamine assessment, and lack of anaesthetic depth monitoring.

The only study measuring nor-adrenaline levels during dexmedetomidine infusion to attenuate the intubation response was carried out in patients undergoing coronary artery bypass grafting. Jalonen et al.^21^ studied the haemodynamic effects of dexmedetomidine, which was administered as a loading dose followed by infusion (50 ng kg^-1 ^minute^-1 ^over 30 minutes before induction of anaesthesia and subsequently 7 ng kg^-1^ minute^-1^ up to the end of surgery), versus placebo in eighty patients.

The authors concluded that, in patients in the study group, dexmedetomidine successfully blunted the intubation response, reduced the intraoperative haemodynamic variability, and decreased the noradrenaline concentration from baseline values without any change in adrenaline values. However, the authors commented that, due to the limited sample size of 40 participants per group, adverse effects such as bradycardia could not be reliably ascertained. Hypotension was more frequent in the study group (9 of 40 patients).

This study differs from the present one in multiple respects. They had administered dexmedetomidine as a higher loading dose, followed by an infusion for the entire duration of the surgery. The sample size was limited; a different patient group was studied; premedication with scopolamine was administered; pancuronium was used as the muscle relaxant; and the study lacked anaesthesia depth monitoring. However, the finding that nor-adrenaline values decreased compared with baseline values, without a change in adrenaline levels, was concordant with ours.

The present study is the only study to date in which attenuation of the haemodynamic pressor response with different doses of dexmedetomidine has been evaluated by assessment of plasma catecholamine levels while ensuring adequate depth of anaesthesia by maintaining BIS between 40 and 60 throughout the study period. Samples for baseline adrenaline and nor-adrenaline levels in all patients were drawn in the pre-operative area because anxiety levels typically rise after transfer to the operating room table, potentially increasing catecholamine concentrations. As the pressor response peaks at around 1-3 minutes of airway manipulation, a second sample was taken at three minutes after endotracheal intubation.

Laryngoscopy and intubation are expected to result in an increase in catecholamine levels.^1, 2^ However, in the present study, there was no rise in the levels of adrenaline or noradrenaline in any of the groups; rather, noradrenaline levels were significantly lower in all three groups three minutes after securing the airway compared with baseline values, while adrenaline levels remained near baseline. In the inter-group comparison, no significant differences were found in the values of adrenaline and noradrenaline. This possibly suggests that all three doses of dexmedetomidine were equally effective in attenuating the catecholamine rise following intubation at an adequate depth of anaesthesia.

In some earlier studies, the increase in adrenaline levels after intubation was observed to be much greater than the increase in nor-adrenaline levels, in some cases up to four times.^22, 23^ Thus, attenuating the increase in the former may be more difficult, which might explain our finding of adrenaline values remaining around baseline, in contrast to the noradrenaline levels observed following intubation with all three doses of dexmedetomidine.

Dexmedetomidine, despite a multitude of benefits, has been associated with adverse effects such as bradycardia and hypotension, especially at doses between 1-2 μg kg^-1^, as reported in the literature.^11, 14, 24, 25, 26, 27, 28, 29^ The observations of the present study are also consistent with regard to the incidence of bradycardia, which was higher in the 1 μg kg^-1 ^group than in the other two dose groups. In two patients belonging to the 1 μg kg^-1 ^group, IV atropine 0.6 mg was administered twice to manage bradycardia; this occurred at the end of the study drug infusion, when the reduction in HR was significantly greater than in the other two groups (0.5 μg kg^-1^ and 0.75 μg kg^-1^).

We found a comparable incidence of hypotension among the three study groups, which was treated by reducing the inhalational agent concentration by 50% when the BIS value was on the lower side of the required range (40-60), and/or by administering IV mephentermine 6 mg. Only 6 patients required administration of IV mephentermine 6 mg as a one-time dose (one, three, and two patients in groups D1, D2, and D3, respectively). The most common time point at which hypotension occurred was 5 minutes post-intubation, possibly because the intubation response had abated, surgery had not yet commenced, and adequate depth of anaesthesia was being maintained.

Contrary to our observations, Silpa et al.,^12^ while comparing 0.5 μg kg^-1^ and 1 μg kg^-1^ of dexmedetomidine for pressor response attenuation, in patients scheduled for elective cardiac surgery found no adverse effects of hypotension or bradycardia with the 1 μg kg^-1 ^dose. Possible reasons for this include the different patient population and a longer duration of dexmedetomidine loading-dose infusion (15 minutes) compared with that in the present study (10 minutes). A few other studies did not observe any adverse effects at the 0.5 μg kg^-1^, 0.75 μg kg^-1^, or 1 μg kg^-1^ doses of dexmedetomidine.^5, 10, 12, 15, 17^

The possible reasons of differences from the observations of the present study could be the choice of induction agent,^5^ the absence of anaesthesia depth monitoring,^5, 10, 12^ glycopyrrolate premedication^15^ and the patient population being studied.^12, 17^

Dexmedetomidine produces a dose-dependent sedative effect and mimics that of natural sleep, with easy arousability.^13, 17^ All doses of dexmedetomidine in the present study showed a decrease in sedation scores and BIS values from baseline compared with values obtained immediately after study drug infusion; however no episode of desaturation (SpO_2_ <95%) was seen in any patient. In contrast, Zhan-Ying et al.^27^ and Sulhyan et al.^30^ observed that a dose of 1 μg kg^-1^ caused desaturation in their studies. In the present study, the induction dose requirement for propofol was reduced with 1 μg kg^-1^ of dexmedetomidine compared with 0.5 μg kg^-1^ and 0.75 μg kg^-1^. This finding differed from that reported by Sharma and Mehta^11^ who concluded that both doses (0.5 and 1 µg kg^-1^) were similar in reducing the propofol dose requirement.

There is an ongoing quest to obtain an optimal dose of dexmedetomidine that results in the desired obtundation of the pressor response, is biochemically confirmed by catecholamine levels, and ensures an adequate depth of anaesthesia. Hence, in the present study, we found that a dose of dexmedetomidine, 0.5 µg kg^-1^, is beneficial, optimal, and cost-effective, with fewer side effects.

### Strengths

The strength of this study lies in its novelty. This is the first study in the literature to assess the optimal dose of dexmedetomidine for obtundation of the haemodynamic pressor response to laryngoscopy and intubation, while maintaining an adequate depth of anaesthesia and including biochemical confirmation by catecholamine level analysis.

### Study Limitations

The possible limitations of the study were, firstly, that it was a single-center study involving patients of Indian origin. Secondly, invasive blood pressure was not monitored, which could probably have provided a more accurate real-time reading. Lastly, since there was no previous study on catecholamine assessment, the sample size was not calculated based on catecholamine levels; this could be addressed in future research on this subject.

## Conclusion

There were no differences among the three doses of dexmedetomidine in attenuating the pressor response to laryngoscopy and intubation, as measured by haemodynamic parameters and catecholamine levels, when adequate anaesthetic depth was maintained. Nevertheless, bradycardia occurred more frequently with the 1 μg kg^-1^ dose, and the 0.75 μg kg^-1^ dose had no added advantage over the 0.5 μg kg^-1^ dose. Hence, 0.5 μg kg^-1^ appears to be the optimum dose of dexmedetomidine for attenuation of the pressor response.

Using catecholamine levels as the primary outcome for sample size calculation would provide greater clarity regarding the optimal dexmedetomidine dose for complete suppression of the stress response.

## Ethics

**Ethics Committee Approval: **It was approved by the Delhi University Faculty of Medicine, Human Research (IEC-HR) Institutional Ethics Committee, (approval no: IECHR-2023-61-12-R2, date: 02.11.2023).

**Informed Consent: **Participants provided written informed consent for this prospective, randomized, double-blind, controlled study.

## Figures and Tables

**Table 1. Demographic Parameters:** 

**Variables**	**Group D1** **(n = 56)**	** Group D2** **(n = 56)**	** Group D3** **(n = 56)**	***P* value**
Age (years)	36.9 (11.1)	36.1 (10.8)	37.7 (12.5)	0.762
Gender (male/female)	43/13	46/10	42/14	0.631
ASA-PS (Ⅰ/Ⅱ)	43/13	48/08	45/11	0.482

Values are represented in mean (standard deviation) or numbers (proportion), D1-dexmedetomidine: 0.5 μg kg^-1^, D2-dexmedetomidine: 0.75 μg kg^-1^, D3-dexmedetomidine: 1 μg kg^-1^

ASA-PS, American Society of Anaesthesiologists Physical Status

**Table 2. Heart Rate (Beats/Minute) Changes at Different Time Points:** 

**Time**	**D1**	**D2**	**D3**	**Inter-group *P* value**
HR at baseline	89.59 (16.48)	88.21 (14.88)	85.48 (14.35)	0.352
HR after study drug completion	80.04* (15.12)	79.45* (16.29)	72.70* (12.21)	0.015# D1 versus D3 (*P*= 0.023) D2 versus D3 (*P*= 0.042) D1 versus D2 (*P*= 0.975)
HR at end point of induction	77.18* (13.41)	76.13* (14.61)	75.02* (11.93)	0.69
HR just before laryngoscopy	77.66* (13.41)	76.39* (14.56)	73.59* (11.78)	0.28
1 minute after intubation	86.05 (10.89)	84.13 (13.91)	82.86 (12.06)	0.389
2 minutes after intubation	80.64* (11.21)	80.50* (11.47)	80.29* (11.11)	0.986
3 minutes after intubation	79.89* (12.55)	78.16* (12.67)	78.02* (9.94)	0.652
5 minutes after intubation	76.52* (12.68)	74.68* (11.38)	77.23* (9.94)	0.425
7 minutes after intubation	76.86* (11.64)	75.00* (13.94)	75.23* (12.41)	0.701

Values are represented in mean (standard deviation) *: *P* value<0.05 compared to baseline: statistically significant, #: *P* value<0.05 between group: statistically significant, D1- dexmedetomidine: 0.5 μg kg^-1^, D2-dexmedetomidine: 0.75 μg kg^-1^, D3- dexmedetomidine: 1 μg kg^-1^

HR, heart rate

**Table 3. Systolic Blood Pressure (mmHg) Changes at Different Time Points:** 

**Time**	**D1**	**D2**	**D3**	**Inter-group *P* value**
SBP at baseline	125.84 (11.6)	129.21 (13.22)	125.57 (11.41)	0.211
SBP after study drug completion	119.73* (19.22)	124.77* (12.82)	122.11* (13.19)	0.22
SBP at end point of induction	108.43* (18.12)	109.91* (14.01)	113.66* (12.31)	0.16
SBP just before laryngoscopy	103.82* (16.34)	106.95* (13.60)	102.52* (17.60)	0.32
1 minute after intubation	121.82 (15.31)	118.29* (16.86)	124.21 (48.46)	0.399
2 minutes after intubation	118.37* (16.57)	113.77* (15.34)	116.45* (13.03)	0.269
3 minutes after intubation	112.75* (17.06)	109.50* (13.77)	110.70* (11.69)	0.490
5 minutes after intubation	107.57* (14.73)	106.50* (12.56)	106.14* (12.22)	0.838
7 minutes after intubation	109.96* (16.35)	106.30* (14.32)	106.77* (14.16)	0.398

Values are represented in mean (standard deviation), *: *P* value<0.05 compared to baseline: statistically significant, D1-dexmedetomidine: 0.5 μg kg^-1^, D2-dexmedetomidine: 0.75 μg kg^-1^, D3-dexmedetomidine: 1 μg kg^-1^

SBP, systolic blood pressure

**Table 4a. Changes in Nor-adrenaline Levels (pg/mL):** 

**Time**	**D1**	**D2**	**D3**	**Inter-group *P* value**
Baseline	8433.6 (4302.8)	9392.0 (7698.2)	9494.1 (5749.5)	0.091
3 minutes after intubation	7685.7 (7710.9)	8207.8 (8043.3)	8448.4 (7111.5)	0.134
Within group *P* value	0.02*	<0.01*	0.02*	-

Values are represented in median (inter quartile range) *: *P* value<0.05 compared to baseline. D1-dexmedetomidine: 0.5 μg kg^-1^, D2-dexmedetomidine: 0.75 μg kg^-1^, D3-dexmedetomidine: 1 μg kg^-1^

**Table 4b. Changes in Adrenaline (pg/mL):** 

**Time**	**D1**	**D2**	**D3**	***P* value**
Baseline	4278.9 (6666.3)	4052.8 (3343.6)	4068.3 (3218.0)	0.362
3 minutes after intubation	4368.8 (5829.5)	3887.9 (3553.7)	3818.9 (3529.3)	0.494
*P* value	0.431	0.712	0.492	-

Values are represented in median-inter quartile range, D1-dexmedetomidine: 0.5 μg kg^-1^, D2-dexmedetomidine: 0.75 μg kg^-1^, D3-dexmedetomidine 1: μg kg^-1^

**Table 5. Percent Change in Catecholamines from Baseline:** 

**Variable**	**D1**	**D2**	**D3**	***P* value**
% change adrenaline	-1.0 (-9.5, 23.4)	-5.5 (-20.0, 17.2)	-4.1 (-12.2, 5.5)	0.412
% change nor-adrenaline	-7.2 (-28.9, 7.8)	-5.2 (-24.3, 5.9)	-6.7 (-22.3, 4.8)	0.993

Values are represented in median-inter quartile range. D1-dexmedetomidine: 0.5 μg kg^-1^, D2-dexmedetomidine: 0.75 μg kg^-1^, D3-dexmedetomidine: 1 μg kg^-1^
